# Researchers’ perspectives on scientific and ethical issues with transcranial direct current stimulation: An international survey

**DOI:** 10.1038/srep10618

**Published:** 2015-06-12

**Authors:** Kate Riggall, Cynthia Forlini, Adrian Carter, Wayne Hall, Megan Weier, Brad Partridge, Marcus Meinzer

**Affiliations:** 1 The University of Queensland, Centre for Clinical Research, Brisbane 4029, Australia; 2 Monash University, School of Psychological Sciences, Melbourne 3168, Australia; 3 The University of Queensland, Centre for Youth Substance Abuse, Brisbane 4029, Australia

## Abstract

In the last decade, an increasing number of studies have suggested that transcranial direct current stimulation (tDCS) may enhance brain function in healthy individuals, and ameliorate cognitive and other symptoms in patients suffering from various medical conditions. This, along with its presumed safety, simplicity, and affordability, has generated great enthusiasm amongst researchers, clinicians, patient populations, and the public (including a growing “do-it-yourself” community). However, discussion about the effectiveness and ethics of tDCS thus far has been confined to small groups of tDCS researchers and bioethicists. We conducted an international online survey targeting the opinions of researchers using tDCS who were asked to rate the technique’s efficacy in different contexts. We also surveyed opinions about ethical concerns, self-enhancement and public availability. 265 complete responses were received and analyzed statistically and thematically. Our results emphasize the potential uses of tDCS in clinical and research contexts, but also highlight a number of emerging methodological and safety concerns, ethical challenges and the need for improved communication between researchers and bioethicists with regard to regulation of the device. Neither the media reputation of tDCS as a “miracle device” nor concerns expressed in recent neuroethical publications were entirely borne out in expert opinion.

New technologies in neuroscience research and their potential application beyond the laboratory have generated significant academic debate over the ethics of their use for human enhancement^1^. Particular interest surrounds the potential use of “non-invasive brain stimulation” (NIBS)^2^ which modulates human brain function, and consequently behaviour, without the risks of invasive surgical intervention or of pharmacological treatments.

In the last decade, an increasing number of studies have suggested that transcranial direct current stimulation (tDCS) may enhance brain function in healthy individuals as well as ameliorating cognitive and other symptoms in patients suffering from various medical conditions^3^,^4^,^5^,^6^. This type of NIBS involves applying a weak electrical current (typically 1-2 mA) via scalp-attached electrodes to influence brain function. These promising findings have produced an enthusiastic response in the media and general public^7^, partly because tDCS is a simple to use, low cost technology that is assumed to be safe due to the apparent absence of serious adverse side-effects^8^,^9^. In addition, tDCS has been promoted as a tool for human enhancement in a growing but so far unregulated “do-it-yourself” (DIY) market for making and using tDCS stimulators^10^.

Along with broader ethical concerns around human enhancement^10^, the tDCS research community is now debating more specific ethical issues about the enhancement use of the technique^11^. These include: its short and long-term safety^12^, the need for greater regulation of public use^1^,^10^, and good research practices in communicating tDCS findings to the public^13^. Some researchers feel that the research community and regulatory bodies must engage with the growing DIY community to ensure safe and responsible use of tDCS^10^. Others have argued that public interest is built largely on hype and misleading communication of results, so interest will quickly dissipate when this enthusiasm proves unfounded^13^. Both, however, emphasise the need for honest and comprehensive communication of findings, be it about safety or the limitations of research studies.

Discussion about the above issues thus far has been confined to relatively few tDCS researchers and bioethicists (see Supplementary Table 1). A recent paper^14^ reported a short survey of the views of researchers in all fields of NIBS (including tDCS) about the utility of and their personal use for neuroenhancement. The study concluded that “the vast majority of specialists on NIBS are reluctant to stimulate their own brains due to insufficient benefit, safety concerns and time required for application”^14^. We have expanded on this survey by seeking the opinions of the international research community on the use of tDCS in the laboratory, the clinic and by the public for enhancement use. We sought their opinions on the efficacy of tDCS in each context, and their concerns about technical and ethical issues raised by its different potential uses.

## Methods

### Participants

The target population for this study were researchers who had published as first or senior authors in the field of human experimental or clinical tDCS research. A pool of participants was gathered from an Endnote database of publications in the field, created by downloading citations from the Pubmed Central and Biosis Reviews databases. Search terms included “tDCS”, “transcranial direct current stimulation”, “transcranial electrical stimulation” and “non-invasive brain stimulation”.

All retrieved publications were reviewed by the first and senior authors to exclude duplicates, animal studies, non-English and non-tDCS related articles. Reference sections of identified reviews were inspected for potentially relevant original articles not retrieved by the initial search. The final dataset comprised 872 articles, dating from 1991-2013. Email addresses of first and senior authors in each of these articles were retrieved from the contact information in the original publications or using publically accessible online directories/databases.

An invitation email was sent to 847 authors that included a short summary of the study and a link where they could access the survey (see below). During this stage, out-dated or incorrect email addresses were manually corrected.

The study was approved by the ethics committee of The University of Queensland. All methods were carried out in accordance with the approved guidelines.

### Online survey

The anonymous online survey was hosted through LimeService (www.limeservice.com). The development of the survey was guided by issues presented in Supplementary Table 1. The participant information sheet appeared as the first page of the survey. After providing consent to participate in the study, participants completed a 24 question survey: The questions addressed age and consent (2 questions), demographic data (4 questions), context of the subject’s use of tDCS (6 questions), opinions on the efficacy of the technique in various contexts (research, clinical and performance enhancement outside of the laboratory, 6 questions), ethical concerns with using tDCS in those contexts (3 questions), and personal use and attitudes towards public use and availability (4 questions). The majority of these were closed questions with room for an optional free-form comment. Space for further comments was provided at the end of the questionnaire.

Questions regarding “efficacy” had 5 response options (0 = ineffective, 1 = partly effective, 2 = mostly effective, 3 = absolutely effective, 4 = no opinion/prefer not to answer). Questions regarding “ethical concerns” had 4 response options (0 = no concerns, 1 = some concerns, 2 = many concerns; 3 = no opinion/prefer not to answer; see Table 1 for details of the survey). The option “no opinion/prefer not to answer” was not included in the statistical analyses.

### Data analysis

Responses to the closed questions were analysed quantitatively. Statistical analyses included descriptive statistics (means/percentages), means comparisons (independent sample t-tests) and predictive statistics (logistic regression analysis), as appropriate to the research question. Several variables were dummy coded for binomial comparisons (e.g., Should tDCS be available to the public; no = 0, yes = 1).

Not all respondents provided qualitative comments as these sections of the survey were optional. Responses to the open questions were analysed thematically^15^ and coded according to the main themes of the survey. Thematic coding covered issues pertaining to: efficacy (N = 304 comments); ethical issues (N = 512); self-use (N = 257); and public use including oversight and regulation (N = 234). Each open comment section was initially coded separately [e.g., statements about the efficacy of tDCS for research (cognition, motor and affect), clinical (neurological, psychiatric) and enhancement purposes]. Individual comments were re-grouped to different sections if they addressed issues pertaining primarily to a different subsection (e.g., comments on clinical effectiveness that were provided in the sections on research effectiveness). Some of the subsections were grouped due to large topical overlap of comments (e.g., effectiveness in different research contexts) and the results summarized to highlight the range of perspectives from tDCS researchers. The qualitative comments were initially coded by one author (KR) and verified by two other authors (CF, MM). Disagreements were settled by discussion until consensus was reached. A response that addressed multiple themes was counted as multiple comments. A response considered identical to a previous comment by the same author was not counted. We present the qualitative data in concert with the quantitative analysis of survey responses in order to better depict the views of respondents.

## Results

### Participants

Demographic characteristics of the 265 participants who completed the survey (31% response rate) are detailed in Table 2. Researchers came from various backgrounds, with the majority being trained in neuroscience (N = 93), psychology (N = 73) or medicine (N = 65). This variable had no effect on the statistical results reported below. At the time of the survey, our sample had a total of 1226 years working experience with tDCS (mean±SD years 4.2 ± 2.9, range 1–22). Participants had published a combined total of 1317 original or review papers on tDCS (5.0 ± 11.5, range 1–119; 61.9% as first or senior author). Approximately half were junior (pre-/post-doctoral fellows, N = 139) and half senior researchers (group leaders/professors; N = 126). Two thirds (64%) described tDCS as central to their research (junior 70%; senior 56%). Senior researchers had more years of experience with tDCS (t(264) = 4.8, p < 0.0001), and had published more papers overall (t(262 ) = 2.9, p = 0.004) or as first or last author (t(255) = 3.7, p = 0.0003).

The focus of tDCS research in our sample was mainly on “cognition” (N = 143), followed by “motor functions” (N = 101) and “affect” (N = 42; multiple answers were allowed). 83 researchers reported other research foci (e.g., pain, tinnitus, methodological aspects).

### Effectiveness of tDCS

Mean effectiveness ratings were highest for research contexts (mean ± SD 1.53 ± 0.73, N = 574 responses), followed by clinical applications (1.50 ± 0.73, N = 304) and enhancement contexts (1.05 ± 0.70, N = 226). Researchers who described tDCS as “central to their research” rated effectiveness higher than those who did not. These effects were statistically significant for all domains except for cognition and motor research (all p = 0.05-0.0004, Supplementary Figure 1A). Senior researchers were more sceptical than junior researchers about the effectiveness of tDCS across all domains (all p < 0.05-0.0009, Supplementary Figure 1B).

**Figure 1:**
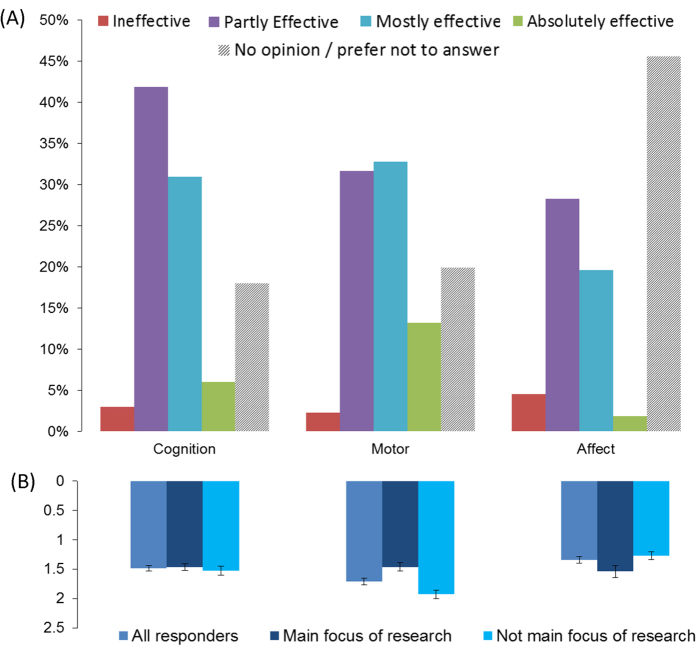
Researchers’ assessment of the efficacy of tDCS in three research contexts according to the general sample (**A**) and focus of research (**B**). Data represents distribution of responses (%) or mean effectiveness ratings for each category, see legend.

The largest proportion of researchers rated tDCS in ***research contexts*** (cognition, motor, affect) as “partly effective” (28-42%) or “mostly effective” (19-33%). Only a small percentage described tDCS as “ineffective” (2-5%) or “absolutely effective” (2-13%; Fig. 1A). Effectiveness ratings were highest for the motor domain (N = 213, mean±SD 1.71 ± 0.77) followed by cognition (N = 218, 1.49 ± 0.69) and affect (N = 143, 1.34 ± 0.68). Effectiveness for “affect” tended to be rated higher by those researchers reporting a research focus in this area (N = 38, 1.53 ± 0.64) as compared to those not working in this area (N = 105, 1.28 ± 0.68, t(141) = −1.95, p = 0.53). Research focus did not affect effectiveness ratings for cognition, but tDCS for motor research was rated as significantly less effective by participants who specialised in motor research than in those who did not (t(211) = 4.64, p < 0.001, Fig. 1B).

In ***clinical contexts***, the distribution of effectiveness ratings was similar to research contexts in the 66% who answered the question. The majority of these rated effectiveness as “partly” or “mostly” effective (neurological: 76%; psychiatric: 55%).

**Figure 2:**
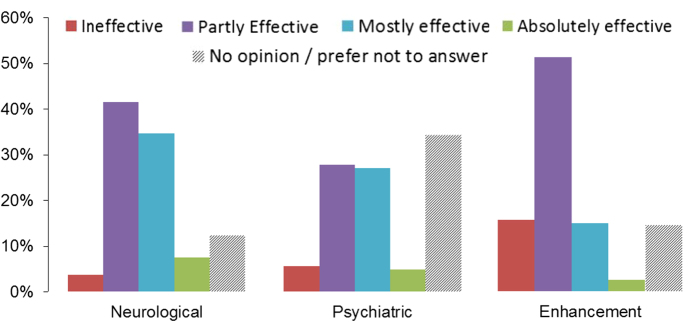
Researchers’ assessment of the efficacy of tDCS in clinical and enhancement contexts. Data represents distribution of responses (%) for each category, see legend.

Only 18% rated effectiveness as “mostly” or “absolutely” effective for ***performance enhancement in healthy individuals*** (52% partly effective; Fig. 2). Overall, a total of 312 comments on the effectiveness of tDCS in various contexts were given. The most common theme in ***research and clinical contexts (N = 238)***, was limitations of the technique (N = 53 comments), namely variability of the effects (N = 24), limited real life relevance due to small effect sizes and the short duration of effects (N = 19), non-focality (N = 7), and other individual problems (N = 11, e.g., blinding issues). The next most common theme was that effectiveness could not yet be determined (e.g., due to the limited number of studies that addressed long-term effects), or that effectiveness may largely depend on the protocol or methodology used (N = 51). This was followed by general comments supporting (N = 49) or questioning (N = 24) the efficacy of tDCS, based on either the current literature or their own findings.

Only 4/66 comments on ***enhancement***, directly supported its efficacy (e.g., citing their own successful experimental work in healthy individuals). Of the remaining comments, 43 were negative, questioning the efficacy of tDCS for enhancement (N = 21), with some suggesting that a healthy brain may already be at an optimum “homeostatic balance” for performance, which limits the potential for benefit to individuals and society. Others expressed uncertainty about efficacy (N = 29) due to factors such as limited real life relevance, variability or short duration of effects.

### Ethical aspects of tDCS use

**Figure 3:**
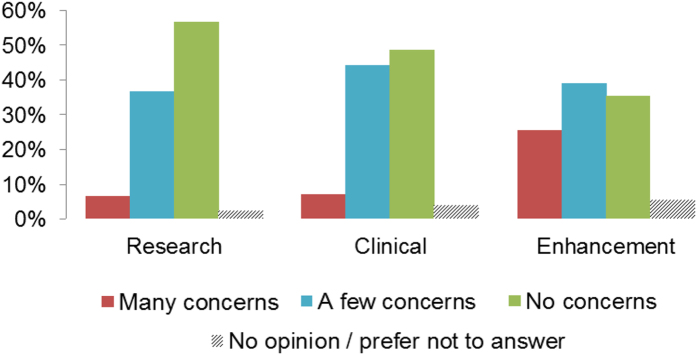
Rating of researchers’ ethical concerns about the use of tDCS on research, clinical and enhancement contexts. Data represents distribution of responses (%) for each category, see legend.

Ethical concerns were more pronounced for enhancement uses of tDCS (mean ± SD 0.90 ± 0.77), than for clinical (0.58 ± 0.62, t(246) = 6.73, p < 0.0001) and research contexts (0.49 ± 0.61, t(246) = 8.33, p < 0.0001). Notably, 25% of participants had “many concerns” regarding “enhancement use of tDCS” (<10% in research or clinical settings, see Fig. 3). Concerns were also more pronounced for clinical than research use (t(246) = 2.34, p = 0.02). The degree of ethical concern did not vary with centrality of tDCS to participant research activities, seniority, research focus or any demographic variables.

Of the 179 comments pertaining to ethical issues in ***research contexts***, the most common concerns were safety issues (N = 70), including lack of research into potential adverse long-term effects. This was followed by concerns about the communication of research findings (N = 35) such as overemphasis on positive results and clinical implications, and the non-reporting of negative results. Concerns were also raised about the weak methodology in some studies (N = 34, e.g., small samples, poor study design, lack of blinding and replications).

In ***clinical contexts***, the most reported concern in the 140 comments was the need for further research (N = 39), with a pressing need for larger randomized clinical trials (N = 35). This was followed by concerns about safety (N = 35) and efficacy of the technique as a treatment (N = 21). The importance of “good practice” in research (e.g., adherence to basic guidelines, administration by trained professionals) was also emphasised (N = 24).

The overwhelming concerns with ***enhancement use***(N = 193) were safety and the risk of side-effects (N = 58) from potentially inappropriate use by non-experts (N = 25). Participants reported that it was not ethical to offer tDCS for enhancement purposes until both its effects and mechanisms were more thoroughly established (N = 34). This was followed by concerns (N = 18) about limited evidence of a favourable risk-benefit ratio.

### Self-use in present sample

The vast majority of participants (219/265) had tried tDCS on themselves. Their primary motivations were “testing of the device” or “training purposes” in research contexts, or feeling that it was “necessary to experience the procedure themselves before trying it on others” (122/126). Only three participants reported personal use for therapeutic purposes: for pain relief, sleepiness, memory or mood enhancement and smoking cessation. Of those who commented further on self-use (N = 63), the majority reported feeling either no (N = 17) or minimal (N = 29) sensation (e.g., mild tingling or itching on the scalp), though some experienced moderately unpleasant effects (N = 11) and one severe effect (chronic migraine).

### Self-use for enhancement

Approximately 30% (N = 81/264) answered that they “would consider using tDCS for self-enhancement” and approximately the same number reported that they “are aware of other researchers” using it for this purpose (24.8%, 68/264). Of the 66 that provided justification, the primary reason for not using tDCS to enhance one’s own performance was lack of real life relevance (N = 7). Others mentioned safety concerns (N = 5), insufficient research evidence for effectiveness (N = 8), and lack of perceived need or interest (N = 8). Some also suggested that they would only consider “enhancement” if there was proof of efficacy or they developed pathological conditions (e.g., stroke, depression) or age-associated functional decline that may benefit from using tDCS (N = 20).

**Figure 4:**
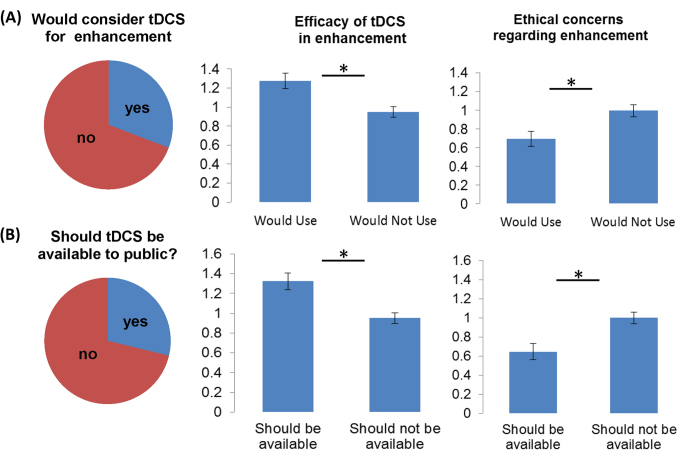
Left column illustrates percentage of researchers who would consider tDCS for enhancement (**A**) and would support public availability (**B**). Middle and right columns show that both aspects are moderated by perceived efficacy for enhancement (middle column) and ethical concerns (right column), * indicate significant differences at p<.05

Logistic regression confirmed that higher perceived effectiveness of tDCS for enhancement and fewer ethical concerns about enhancement use predicted preparedness to use tDCS for self-enhancement (Fig. 4A). The whole model test was significant (χ^2^(4) = 20.19, p < 0.001). Participants who believed tDCS was effective for enhancement had 1.68 higher odds (95% CI = 1.08-2.61) of being willing to use tDCS on themselves for enhancement (Wald(1) = 5.27, p = 0.022), whereas participants who indicated more ethical concerns with using tDCS for enhancement were 0.59 times less likely (95% CI = 0.41-0.89) of indicating that they would consider using it on themselves (Wald(1) = 6.48, p = 0.011). Neither seniority nor centrality of tDCS to research yielded significant differences in these odds (Wald(1) = 0.89-1.11).

### Availability to the public (Fig. 4B)

71% percent of researchers in our sample believed that tDCS should not be available to the public. Logistic regression analysis showed that this was predicted by beliefs about effectiveness and ethical concerns (χ^2^(4) = 19.53, p = 0.001). Participants who believed tDCS was effective for enhancement had 2.00 times higher odds (95% CI = 1.27-3.19) of supporting tDCS being available to the public (Wald(1) = 8.80, p = 0.003). Participants who had more ethical concerns with using tDCS for enhancement were 0.62 times less likely (95% CI = 0.41-0.92) to indicate that it should be available to the public (Wald(1) = 5.55, p = 0.018). Again here, seniority or centrality did not yield significant differences in these odds (Wald(1) = 0.24-0.70).

Only 7 of 234 comments supported tDCS being made available to the public. Reasons provided were that “restriction of sale may fuel underground or DIY use”, “it would be difficult to justify restrictions legally in the context of free availability of other neuroenhancers”, and “that restrictions would limit free enterprise and autonomy”. Some researchers (N = 15) would accept commercial sale but only with appropriate regulation “to ensure quality control of the device and appropriate application parameters including duration, strength, frequency and electrode positioning”. Of those who argued against commercial sale of tDCS, the most common concern was lack of understanding of the underlying mechanisms and potential risks or need for further research regarding efficacy and “clinical” outcomes (N = 63). Safety (N = 45) and misuse by “untrained” non-experts (N = 50) were the next greatest concerns. Specifically, there were calls for lay users to be required to have “detailed knowledge of the technique and potential risks like overdosing” or “close supervision and oversight by trained professionals”.

## Discussion

The increase of interest in tDCS in research, clinical an public contexts has spurred a number of recent opinion papers on potential methodological limitations and ethical issues and calls for better oversight and regulation (Supplementary Table 1). These publications only represent the opinions of small groups of researchers. Our study describes the views of a large sample of junior and senior tDCS researchers with different training backgrounds and from different research areas. Our data present the perspectives of researchers directly involved in using this technique who will be responsible for shaping the future of tDCS research.

### Effectiveness of tDCS

The majority of researchers who participated in this survey presented a cautious evaluation of the efficacy of tDCS. tDCS was characterised as partly or mostly effective in research and clinical settings with few responses describing it is as “ineffective” or “absolutely effective”. Researchers balanced their optimistic perspective of efficacy with comments discussing current limitations that preclude definitive conclusions about effectiveness at this stage about long-term effects, real-world transfer and potential clinical applications. Indeed, the need for further research was addressed in almost every section of this survey in a total of 149 unique comments. The cautious approach to use of tDCS is also reflected in the low ratings for efficacy of its use to enhance normal functions. The tDCS research community appears well aware of current limitations and the need for future research to address those. The appearance of enthusiastic articles in the media and online^7^ suggest that these cautious views are frequently not being communicated clearly to the public. Researchers must make their views about the limited evidence of safety and efficacy clearly when speaking with the media or communicating with the public.

A very different picture emerged in participants’ views about use of tDCS to enhance normal functions. Overall ratings were substantially lower than for use in research and clinical contexts. In addition, a relatively high percentage of researchers perceived tDCS as “ineffective” in this context and only four comments directly supported its efficacy.

Effectiveness ratings varied with domain. Effectiveness was rated highest in the motor domain but researchers who reported a research focus in this area rated effectiveness significantly lower than researchers who did not. This observation is mirrored by recent studies highlighting methodological and theoretical issues in this domain^11^,^16^,^17^. Moreover, across domains, researchers describing tDCS as central to their research rated effectiveness higher than those who did not. A similar pattern was found for junior researchers. Tentatively, this may reflect the direct experience of those researchers with the effectiveness of tDCS as a research and/or clinical tool, but may also point to a potential bias among those building their (emerging) careers on this technique. It could also reflect senior researchers’ greater experience with previously highly promised technologies that failed to be realised, and therefore greater caution in this case.

### Ethical issues

The most pronounced ethical concerns across the sample were expressed for enhancement contexts. This did not vary with seniority, centrality and research focus, thereby highlighting the ubiquity of these concerns in the tDCS community. The most common concerns related to safety including a limited risk-benefit ratio when using tDCS for enhancement of “normal” brain functions in healthy individuals. Key safety concerns mainly overlapped with those expressed in previous publications (Supplementary Table 1) emphasizing the need for further research into these issues.

Even though ethical concerns were less pronounced for research, a large number of comments addressed inappropriate research methodology and poor communication of research results. In clinical use, there were concerns about the need for larger controlled clinical trials and better research on potential long-term adverse effects. The latter knowledge gap was perceived to be particularly critical in the light of recent evidence that enhancement of a targeted function may result in potentially detrimental effects on non-targeted brain functions^18^. Interestingly, this concern contrasts with the perceived limited effect sizes of favourable tDCS effects in research and clinical settings. Nonetheless, it must be acknowledged that the good reputation for safety enjoyed by tDCS has come mostly from studies that assessed short-term stimulation effects and very little is known about the potential long-term effects of repeated stimulation sessions^12^ or its potential maladaptive effects on plasticity when tDCS is combined with behavioural interventions^9^. Therefore, calls for further research into long-term effects of tDCS and its interaction with treatment are warranted.

While safety issues appeared to be a major concern among tDCS researchers, few comments addressed the lack of safety recommendations derived from expert consensus for tDCS such as those published for other NIBS techniques^19^. Indeed, while general recommendations and discussions about appropriate stimulation parameters had been published at the time of this survey^20^,^21^,^22^, a set of “standard safety parameters” have only recently been outlined that can now be applied and tested in different contexts^9^.

### Self-use in the present sample

The majority of researchers who participated in this survey had used tDCS on themselves, mainly to experience the stimulation and possible adverse effects before using it in research. This is in line with a previous study that explored the self-use of NIBS in scientists^14^. Of those who commented on their experience, the majority reported only minimal side effects and adverse effects reported were similar to that in previous literature reviews^8^,^9^.

Surprisingly few participants reported self-use for enhancement or therapeutic purposes. However, approximately 30% stated that they would use it for self-enhancement or treatment if deemed necessary (e.g., to address pathological conditions in the future). This emphasizes that the research community perceives tDCS primarily as a potential therapeutic tool than as a device to enhance “normal” brain functions. Unsurprisingly, those who perceived tDCS to be more effective (and reported fewer ethical concerns) were more likely to consider using it for enhancement purposes which mirrors the recent media hype and also the interest and hopes of the DIY community^7^.

### Availability to the public

Even though the vast majority of researchers were opposed to public availability (>70%), very few comments discussed ways to regulate tDCS (N = 15/234). Therefore, the tDCS research community appears to be more concerned with “research outcomes”, including methodological issues, effectiveness and safety, rather than discussions of how to regulate these devices. This is also illustrated by the fact that a recent opinion paper outlining the regulatory status of tDCS by tDCS experts did not cite any papers favouring regulation published by bioethicists^1^,^10^. Interdisciplinary communication needs to replace parallel discussions of these issues so researchers can take a more active role in guiding the translation of the technology into other settings and helping to create regulations that will protect the public from the misuse of these devices.

## Conclusions

This comprehensive survey provides the first snapshot of the tDCS research community opinion on its use. Our results emphasize the potential of tDCS in clinical and research contexts, but also highlight a number of unresolved issues, including methodological and safety concerns, ethical challenges. The disjuncture between these views and discussions in the bioethics literature suggests the need for improved interdisciplinary communication. Our results indicate that tDCS researchers do not support either the media portrayal of tDCS as a “miracle device” nor the major concerns expressed in recent neuroethical publications.

## Additional Information

**How to cite this article**: Riggall, K. *et al.* Researchers' perspectives on scientific and ethical issues with transcranial direct current stimulation: An international survey. *Sci. Rep.* **5**, 10618; doi: 10.1038/srep10618 (2015).

## Supplementary Material

Supplementary Information

## Figures and Tables

**Table 1:** Survey questions and number of responses to survey questions on effectiveness of tDCS and ethical concerns for its use in research, clinical and enhancement contexts (O = optional questions, M=mandatory questions, N = number of responses, closed questions)

**Question**	**N**	**Response options (score)**
**Effectiveness**		
** How effective is tDCS (M)**		
** **…in research - cognition	218	Ineffective (0)
** **…in research - motor	213	Partly effective (1)
** **…in research - affect	143	Mostly effective (2)
** **…in clinical – neurological	232	Absolutely effective (3)
** **…in clinical – psychiatric	173	No opinion / prefer not to answer
** **…in enhancement	226	
**Ethical**		
** Ethical concerns (M)**		
** **… in research	259	No concerns (0)
** **… in clinical	255	A few concerns (1)
** **… in enhancement	251	Many concerns (2)
		No opinion / prefer not to answer
**Use and availability**		
** Have you tried tDCS on yourself (O)**	265	Yes, No
** Would you consider it for self-enhancement)? (O)**	264	Yes, No
** Are you aware of others using it? (O)**	264	Yes, No
** Should tDCS be available to public (O)**	264	Yes, No

**Table 2:** Summary of the demographic information of survey participants (O = optional questions, M = mandatory questions, N=number of responses).

**Question/parameter**	**Answer options**	**Range, mean ± SD or sum**	**N**
**Age (O)**	open	23-69, 38.8 ± 9.3	255
**Sex (O)**	female, male	87, 171	257
**Position/seniority (M)**	Pre-doctoral researcher (junior)	42 (Junior 139)	265
	Post-doctoral researcher (junior)	75	
	Senior research fellow (senior)	83 (Senior 126)	
	Other	65	
**Training (O)**	Medicine	66	265
	Psychology	73	
	Neuroscience	92	
	Other	34	
**tDCS central to research (O)**	yes, no	169, 95	264
**Years working with tDCS (O)**	Open	1-22, 4.6 ± 2.9	265
			
**# journal articles (O)**			
** **…in total	Open	0-119, 5.0 ± 11.5	263
** **…as first or last author	Open	0-75, 3.2 ± 6.5	257
**Focus of research (O) **(options: select 1 or more)	Cognition	143	265
	(incl. language, memory & attention)		
	Motor function	101	
	Affect (emotion, mood & motivation)	43	
	Other	81	
