# Supplementary material for: Researchers’ perspectives on scientific and ethical issues with transcranial direct current stimulation: An international survey
Source: Sci Rep. 2015 Jun 12;5:10618. doi: 10.1038/srep10618 (PMC4464285; doi:10.1038/srep10618)
Supplement: Supplementary Information [file srep10618-s1.pdf]

Researchers' perspectives on scientific and ethical issues with transcranial direct current stimulation: An international survey

Kate Riggall, Cynthia Forlini, Adrian Carter, Wayne Hall, Megan Weier, Brad Partridge, Marcus Meinzer

Supplementary Figure 1: Moderating effects of centrality of research and seniority on effectiveness ratings of tDCS (y-axis) in research, clinical and enhancement contexts (* indicate significant differences at p<.05, error bars represent standard errors of the mean).

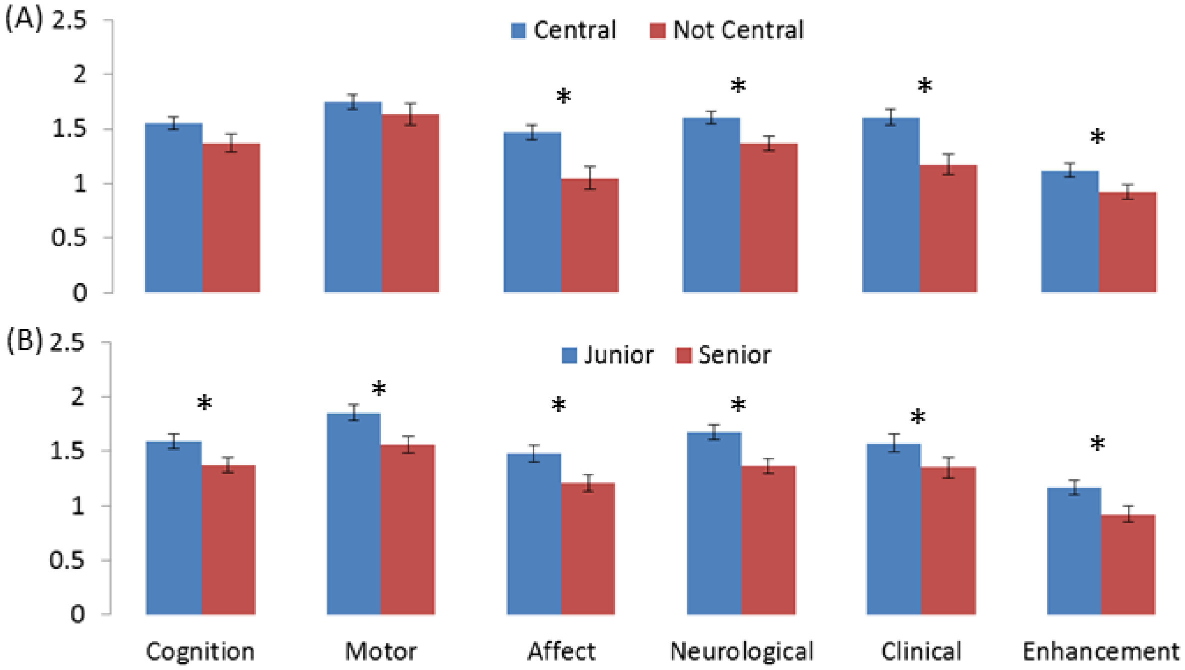

Supplementary Table 1 Examples of scientific, ethical and policy issues discussed in current academic literature on tDCS

**Methodology and Efficacy**	**Variability**: Potentially high variability in tDCS response, both within and between subjects [1]
	**Polarity**: There may be a more complex relationship between electrode polarity and cortical excitability than initially proposed and the current polarity may change over time, notably during longer stimulation [1]
	**Blinding**: Blinding is not assessed in many studies and may not be effective at higher stimulation intensities [1, 2]
	**Current variations**: Current intensity and distribution is influenced by a number of factors: e.g., active and reference electrode placement, shunting of current, or TMS coil placement reliability in motor tDCS studies. Both may also be affected by inter-individual differences in anatomy, neurophysiology and psychological state [1]
	**Spatial specificity**: Large electrodes typically used for tDCS may impact neighbouring brain regions and large scale functional networks may be affected including regions distant from the targeted stimulation site [3, 4, 5]
	**Real life relevance**: Small effect sizes in highly controlled experimental contexts may not translate into noticeable effects outside the laboratory or to "real life" outcomes for subjects [5, 6, 7]
	**State dependency and interference**: Brain stimulation effects may interact with medications or be state dependent. For example, concurrent motor or cognitive tasks may interfere with amplitude modulation of motor evoked potentials in motor tDCS studies [1, 2]
**Ethical issues**	**Safety**: There is evidence of brain damage from tDCS in animal models which may also occur in humans when not used according to "guidelines". Safety may not be guaranteed outside of controlled laboratory contexts (e.g., above critical current limits and/or duration, [2, 7, 8]) Limited knowledge regarding the potential detrimental effects of repeated stimulation in the short and long-terms [2, 3, 5, 7, 8, 9] Perceived safety may lead users to push limits beyond what has been described in literature as "safe" (e.g. higher intensity, longer stimulation duration) and DIY stimulators may cause more serious side effects than commercially available stimulators [8, 10, 11] Questions about whether it is appropriate to use tDCS on the developing brains of minors [6, 7]
	**Enhancement**: Issues pertaining to enhancement of "normal" brain functions (cosmetic neurology, [10]) that change a 'patient-client" relationship into a "buyer-client" relationship Acceptability of tDCS stimulation changes whether it is perceived as analogous to performance-enhancing drugs or a tool that "primes" the brain to learn [6, 10]Despite similar safety and efficacy concerns to other medical devices, the DC stimulator would not be regulated as a medical device if intended for enhancement [7, 11]
	**Personal choice/Coercion**: Potential for forced use (e.g., by employment contracts) either for good (e.g., enhance vigilance of air traffic conductors, law enforcement to detect lies) or bad (e.g., military contexts[5, 10])
	**Self and personhood**: tDCS could modify intrinsic personality characteristics such as empathy and social behaviour, thereby, potentially causing harm to others (enhancement of antisocial traits in business contexts, [5, 10])
	**Equality and justice**: In a society where use of tDCS is encouraged, inequalities in access may emerge [10]
	**Addiction**: Certain uses of tDCS could enable behavioural addictions (e.g. gaming and gambling, [4])

**Oversight**	**Regulation of the device:** In some countries tDCS is regulated as a “medical device” specifically for clinical use. Currently limited commercial regulation or oversight exists, yet devices are being sold to the public [3, 7, 8, 12]. tDCS devices are perceived as “non-invasive”, therefore less problematic than pharmaceutical or surgical interventions, leading to more common use [6]
	**Commercialization**: tDCS devices are relatively inexpensive, easy to access, build and commercialise [3, 6]. There may be a need for consumer protection in an open market [2]
	**Guidelines for professionals**: Ethical guidelines and training may be necessary for physicians who are responsible for advising and treating patients to ensure appropriate and safe use [2, 3, 4, 6, 7]
	**Regulation for public use**: In the present context, only the sale and purchase of tDCS stimulators can be regulated, not the use [2]. Use of tDCS by the public might lead to unexpected innovation [2, 3, 4, 7, 8, 11]. Current laws in the US and Europe could allow tDCS devices to be marketed freely [12]. There is a need for international coordination of policies [7, 11]
**Communication**	**Hype**: Potential confusion due to undue emphasis of benefits over the methodological limitations of tDCS [2, 4]<br>Media optimism may shape public views about tDCS or even influence risk-benefit perception and impact public use as well as patient consent in clinical trials [7]
	**Common misconceptions about tDCS**: Several misconceptions are carried forward in the communications of study results (e.g., the mechanism of tDCS is simple and well understood, it has been used effectively for a long time, there is little or no risk involved with enhancement; [7])<br>Described as non-invasive, yet electrical currents do go through the brain and may cause collateral damage or effects [11]

References

1. Horvath JC, Carter O, Forte JD. Transcranial direct current stimulation: five important issues we aren't discussing (but probably should be). *FrontSyst Neurosci* **8**, 2 (2014).

2. Walsh VQ. Ethics and social risks in brain stimulation. *Brain Stim* **6**, 715-717 (2013).

3. Fitz NS, Reiner PB. The challenge of crafting policy for do-it-yourself brain stimulation. *J Med Ethics*, (2013).

4. Santarnecchi E, Feurra M, Galli G, Rossi A, Rossi S. Overclock your brain for gaming? Ethical, social and health care risks. *Brain Stim* **6**, 713-714 (2013).

5. Sehm B, Ragert P. Why non-invasive brain stimulation should not be used in military and security services. *Front Hum Neurosci* **7**, 553 (2013).

6. Cohen Kadosh R, Levy N, O'Shea J, Shea N, Savulescu J. The neuroethics of non-invasive brain stimulation. *Curr Biol* **22**, R108-111 (2012).

7. Dubljevic V, Saigle V, Racine E. The rising tide of tDCS in the media and academic literature. *Neuron* **82**, 731-736 (2014).

8. Bikson M, Bestmann S, Edwards D. Neuroscience: transcranial devices are not playthings. *Nature* **501**, 167 (2013).

9. Hamilton R, Messing S, Chatterjee A. Rethinking the thinking cap: ethics of neural enhancement using noninvasive brain stimulation. *Neurology* **76**, 187-193 (2011).

10. Lapenta OM, Valsek CA, Brunoni AR, Boggio PS. An ethical discussion of the use of transcranial direct current stimulation for cognitive enhancement in healthy individuals: a fictional case study. *Psychol Neurosci* **7**, 175-180 (2014).

11. Maslen H, Douglas T, Cohen Kadosh R, Levy N, Savulescu J. The regulation of cognitive enhancement devices: extending the medical model. *J Law Biosci* **1**, 68-93 (2014).

12. Fregni F, *et al.* Regulatory considerations for the clinical and research use of transcranial direct current stimulation (tDCS): Review and recommendations from an expert panel. *Clin Res Regul Affairs*, (2014).
